# Characteristics of the deventilation syndrome in COPD patients treated with non-invasive ventilation: an explorative study

**DOI:** 10.1186/s12931-022-01924-y

**Published:** 2022-01-21

**Authors:** Mareike Lüthgen, Stephan Rüller, Christian Herzmann

**Affiliations:** 1 Research Center Borstel, Leibniz Lung Center, Center for Clinical Studies, Borstel, Germany; 2 Research Center Borstel, Leibniz Lung Center, Early Life Origins of Chronic Lung Disease, Borstel, Germany

**Keywords:** Deventilation syndrome, Morning dyspnea, Non-invasive ventilation, NIV, Ventilatory insufficiency, Hypercapnic respiratory failure, COPD, High-intensity NIV, PLBV

## Abstract

**Background:**

Non-invasive ventilation (NIV) is a recommended treatment for COPD patients suffering from chronic hypercapnic respiratory failure. Prolonged dyspnea after mask removal in the morning, often referred to as deventilation syndrome, is a common side effect but has been poorly characterized yet. This study aimed to explore the pathomechanism, identify risk factors and possible treatment strategies for the deventilation syndrome.

**Methods:**

A prospective, controlled, non-blinded study was conducted. After a night with established NIV therapy, the patients underwent spirometry, blood gas analyses and 6-min walking tests (6MWT) directly, at 2 and 4 h after mask removal. Dyspnea was measured by the modified Borg scale. Bodyplethysmography and health-related quality of life (HRQoL) questionnaires were used. Patients suffering from deventilation syndrome (defined as dyspnea of at least three points on the Borg scale after mask removal) were treated with non-invasive pursed lip breathing ventilation (PLBV) during the second night of the study.

**Results:**

Eleven of 31 patients included (35%) met the given criteria for a deventilation syndrome. They reported significantly more dyspnea on the Borg scale directly after mask removal (mean: 7.2 ± 1.0) compared to measurement after 2 h (4.8 ± 2.6; p = 0.003). Initially, mean inspiratory vital capacity was significantly reduced (VCmax: 46 ± 16%) compared to 2 h later (54 ± 15%; p = 0.002), while no changes in pulse oximetry or blood gas analysis were observed. Patients who suffered from a deventilation syndrome had a significantly higher mean airway resistance (Reff: 320 ± 88.5%) than the patients in the control group (253 ± 147%; p = 0.021). They also scored significantly lower on the Severe Respiratory Insufficiency Questionnaire (SRI; mean: 37.6 ± 10.1 vs 50.6 ± 16.7, p = 0.027). After one night of ventilation in PLBV mode, mean morning dyspnea decreased significantly to 5.6 ± 2.0 compared to 7.2 ± 1.0 after established treatment (p = 0.019) and mean inspiratory vital capacity increased from 44 ± 16.0% to 48 ± 16.3 (p = 0.040).

**Conclusions:**

The deventilation syndrome is a serious side effect of NIV in COPD patients, characterized by increase of dyspnea. It is associated with decrease in vital capacity, exercise tolerance after mask removal and lower HRQoL. Patients with high airway resistance are at greater risk of suffering from morning dyspnea. Ventilation in PLBV mode may prevent or improve the deventilation syndrome.

*Trial registration*: The study was registered in the German Clinical Trials Register (DRKS00016941) on 09 April 2019.

## Background

COPD is one of the leading causes of death worldwide [1]. In severe disease, patients often suffer from hypercapnic respiratory failure and treatment with non-invasive ventilation (NIV) is strongly recommended [2]. Studies have shown that among other benefits, NIV can reduce mortality, improve blood gases, and improve quality of life [3–5]. However, positive effects of NIV are proven in patients with stable hypercapnia who are ventilated with high-intensity NIV (HINIV). HINIV uses high inspiratory pressures up to 30 mmHg to achieve a maximum of pCO_2_ reduction. It is often combined with high respiratory rate back-up rates [2, 6]. A common side-effect of HINIV is the so-called deventilation syndrome, i.e. morning dyspnea immediately after removing the ventilation mask that can last several hours. This impairs the patient’s daily activities, reduces HRQoL and therapy compliance. Although approximately 30% of NIV-patients with COPD may suffer from the deventilation syndrome [7], the underlying mechanism remains unclear. A correlation between patient-ventilator-asynchrony, Auto-PEEP-phenomena and morning dyspnea has been described [7, 8]. This suggests that dynamic hyperinflation, exacerbated by high inspiratory pressure may cause the deventilation syndrome [7]. Other explanations discussed are chronic muscle fatigue after rapid cessation of positive ventilation pressure or poor sleeping patterns [9]. In addition to optimizing ventilation settings [7], a potential therapy for morning dyspnea could be ventilation in pursed-lips-breathing-ventilation (PLBV) [8]. This ventilation mode is characterized by a dynamic increase in pressure during the expiratory phase followed by a slow pressure decay, which could prevent airway collapse and thus lead to more adequate expiration and less air trapping. The aim of this study was to explore clinical parameters associated with the deventilation syndrome to identify possible underlying mechanisms, risk factors and strategies which may prevent its occurrence.

## Methods

### Study design

We conducted a prospective, controlled, single centre, non-randomized, descriptive trial with an interventional element (PLBV) during the second night. Investigations were performed from August 2019 until February 2021. The deventilation syndrome was defined by the following two criteria based on expert consensus in the recruiting center: (1) subjective complaints of worsening dyspnea after ventilator mask removal and (2) patient reports three or more points on the Borg scale after ventilator mask removal. The patients were stratified into two groups during the study: (1) patients with deventilation syndrome, (2) patients without deventilation syndrome (control group).

Approval was obtained from the Ethics Committee of the University of Lübeck, Germany, reference 19-025. The study was registered in the German Clinical Trials Register (DRKS00016941).

### Patients

Patients were enrolled when the following criteria were fulfilled: (1) patient has COPD, (2) bilevel mode (inspiratory pressure > expiratory pressure) was applied, (3) patient understood the study requirements, (4) patient was physically able to participate in the study examinations. Exclusion criteria were: (1) acute exacerbations of COPD, (2) pneumothorax 3) invasive ventilation 4) patient is unable to perform the study investigations.

### Study protocol

The investigations were performed on two and three consecutive days in patients without and with deventilation syndrome, respectively. Study subjects spent all nights in the sleep laboratory. On the first day, baseline measurements were performed in the afternoon. On the second and third day, the investigations were conducted immediately when the patients took off their ventilation masks. Patients were therefore asked to wear the mask until the study personnel arrived. Dyspnea and pulse oximetry were reported every 30 min starting directly after mask removal. Spirometry and inspiratory flow were measured directly, after 2 and 4 h. 6-min walking test and blood gas analysis were conducted directly and after 4 h. After mask removal, after 2 and 4 h the measurements were done in the following order: first measurement of dyspnea and pulse oximetry, second blood gas analysis, third spirometry and at last 6-min walking test. If no deventilation syndrome was reported, the study was terminated on the second day, as no changes in respiratory quality were suspected. If the patients reported a deventilation syndrome, they were ventilated in PLBV mode during the whole second night and investigations were repeated thereafter.

### Measurements

Demographic data, COPD stage according to the Global Initiative on Lung Disease (GOLD), comorbidities, current medication, long-term oxygen therapy and ventilation parameter were documented. Pulse rate and oxygen saturation were measured by pulse oximetry. Capillary blood gas analyses were taken and measured using the ABL 90 blood gas analyzer (Radiometer, Germany). Patients received their usual rate of oxygen supply during blood sampling. Spirometry was performed with the mobile spirometer Flowscreen V2.2.4 (Jaeger, Germany). Inspiratory airflow was measured by the In-Check-Dial G16^®^ (Clement Clarke, UK) which was originally developed to measure the capability of inhalator-use. With one forced inspiration breath, patients can move a plastic sail on a scale from 0 to 120 ml in variable difficulty levels. Only the easiest level was used for our examinations, requiring the least inspiratory flow. Bodyplethysmography was performed with the Masterscreen (Jäger/CareFusion, Germany). The 6-min walking test was performed on a straight horizontal 30-m track. Oxygen saturation and dyspnea on the Borg scale were reported before and after exercise. Muscle strength was measured using a 3 kg dumbbell. Patients lifted and lowered it with their dominant arm in sitting position from their knee to their shoulder following the beat of a metronome at 40 bpm for as long as possible. The total time and the reason for stopping (dyspnea, muscle or joint pain) were documented. At night, patients underwent polysomnography, with recording of EEG, EMG, EKG, oxygen saturation, pulse, breathing rate, respiratory flow and ventilator pressure. Dyspnea was assessed using the modified Borg scale. Lastly, the patients answered the validated SRI [10] and CAT [11] questionnaires.

### Intervention

In patients suffering from deventilation syndrome in the morning of the second day, pursed-lip-breathing ventilation (PLBV) using the Vigaro^®^ ventilator (FLO Medical Technologies, Germany) was established for the second night. The PLBV mode imitates the pursed lips breathing which is actively performed by many patients during the day. During this maneuver, there is initially an increase in pressure during the expiration phase, which then slowly decreases to the expiration level. In PLBV mode, the pursed lips breathing delay (PLBD) and pursed lips breathing pressure (PLBP) are set in addition to usual settings in bilevel mode. Compared to conventional ventilation, the inspiratory ventilation pressures can usually be reduced while maintaining CO2 values. At higher IPAP values, PLBD and PLBP are usually also set higher. The setting of PLBV mode was made together with the patients in the afternoon before the third night [8].

### Sample size

Due to a lack of data about the deventilation syndrome, the sample size was calculated based on clinical experience. For an estimated change from seven points on the Borg scale to four points with a standard deviation of three points, at least ten patients were needed at a two-sided level alpha of 0.05 with a power of at least 80%. About 30% of all non-invasively ventilated patients suffer from a deventilation syndrome [7], resulting in a total of at least 30 patients that had to be enrolled. As the calculation of the sample size was based on estimates, a sample size of n = 40 was planned.

### Statistical analysis

Normal distribution was measured by the Shapiro–Wilk test. An unpaired t-test was performed to compare the means of the two groups. A paired t-test was performed to compare the means of the morning measurements and the means of the two ventilation modes. If no normal distribution was given, a Mann–Whitney U test was performed for unpaired samples or a Wilcoxon rank test for paired samples. The measurements directly after mask removal were compared to the following measurement that was performed after 2 h (dyspnea, spirometry) or 4 h (blood gas analysis, 6-min walking test). Further, the measurements after established ventilation were compared to ventilation after ventilation in PLBV mode. The significance level was set at p = 0.05.

## Results

### Drop-outs

**Fig. 1:**
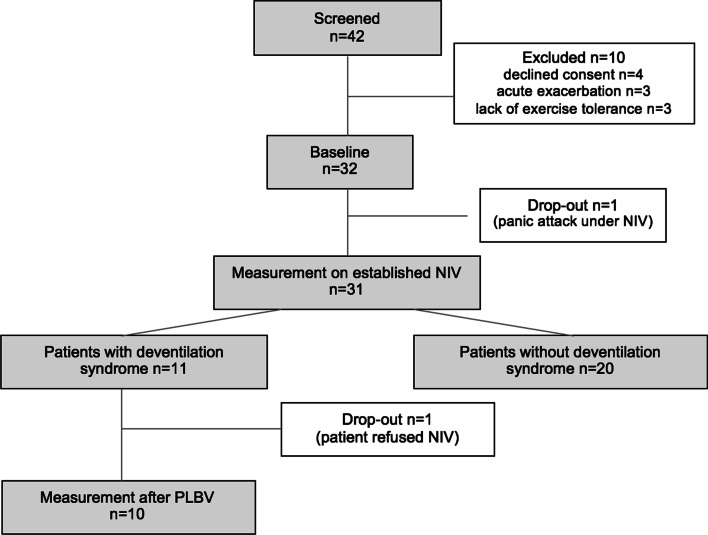
Study flow chart

The recruitment of study subjects was incomplete due to (a) disruptions of clinical service between March 2020 and November 2021 due to the COVID-19 pandemic and (b) final closure of the recruiting hospital due to financial reasons in November 2021. Forty-two long-term non-invasively ventilated COPD patients were screened (Fig. 1). Ten did not meet the inclusion criteria or declined consent. Thirty-two patients underwent the baseline examination, of whom one experienced a panic attack in the first night due to massive dyspnea and discontinued the study. Of the 31 remaining, 11 patients had a deventilation syndrome according to the above criteria, 20 did not. Of the 11 patients with deventilation syndrome, one patient refused further NIV therapy. The ventilation of 10 was switched to PLBV mode and examined on day three.

### Baseline measurements

Baseline data for the two groups were measured in the afternoon (Table 1). There were no group differences in age, medication, comorbidities, sleep parameters, dependence of long-term-oxygen, oxygen saturation at rest, and severity or number of exacerbations of COPD in the last year. Patients who suffered from morning dyspnea had a significantly lower mean body mass index (BMI) (22.5 ± 5.1) than those who did not (27.6 ± 6.5; p = 0.032). In addition, patients with deventilation syndrome had significantly lower mean inspiratory vital capacity (VCmax: 51 ± 16.9% vs 67 ± 12.2%, p = 0.015), FEV1 (29 ± 14.6% vs 38 ± 11.8%, p = 0.001) and higher airway resistance (Reff: 320 ± 88.5% vs 253 ± 147%, p = 0.021). No significant difference in inspiratory flow was observed. Functionally, patients with deventilation syndrome had a worse exercise tolerance than the control group, with a significantly shorter mean walking distance in the 6MWT (149.9 ± 112.4 m vs 236.7 ± 112.8, p = 0.049). The muscular exercise capacity also tended to be reduced, but was not significantly different.

**Table 1:** Baseline demographic data and clinical characteristics

	Patients with deventilation syndrome (*n* = *11*)	Patient without deventilation syndrome (*n* = *20*)	*p*
Baseline data			
Sex m/f (%)	55/45	35/65	
Age, years	68.5 (6.5)	69.9 (6.6)	0.573
BMI (kg/m^2^)	22.5 (5.1)	27.6 (6.5)	
Ventilator settings			
Duration of NIV (months)	30.7 (38.0)	21.3 (23.6)	0.509
Inspiratory pressure (mmHg)	16.7 (4.9)	16.7 (3.3)	0.999
Expiratory pressure (mmHg)	5.6 (1.2)	6.7 (1.4)	0.051
Respiratory rate	11.78 (3.08), n = 2: none set	13.67 (1.84), n = 8: none set	
Ti max (s)	1.42 (0.24)	1.21 (0.23)	**0.037**
Ti min (s)	0.86 (0.21)	0.80 (0.24)	0.461
Blood gas analysis			
pH	7.41 (0.02)	7.40 (0.03)	0.302
pO_2_ (mmHg)	63.3 (12.0)	63.8 (13.2)	0.914
pCO_2_ (mmHg)	47.0 (9.7)	46.7 (7.0)	0.913
Pulmonary function *(n* = *28)*	*n* = *10*	*n* = *18*	
VCmax (l)	1.56 (0.37)	1.98 (0.50)	**0.016**
VCmax (%)	51 (16.9)	67 (12.2)	**0.015**
FVC (%)	48 (16.3)	62 (12.8)	
FEV1 (%)	29 (14.6)	38 (11.8)	**0.001**
FEV1/FVC	58 (10.2)	66 (14.2)	0.148
TLC (%)	136 (20.1)	124 (31.8)	0.089
RV (%)	276 (61.8)	218 (81.5)	0.060
RV/TLC	78 (5.5)	69 (7.1)	**0.001**
Reff (%)	320 (88.5)	253 (147)	**0.021**
SReff (%)	627 (165.6)	395 (165.6)	**0.006**
Rtot (%)	412 (138.3)	299 (169.4)	**0.012**
Inspiratory flow (ml)	68.2 (16.6)	72.0 (12.8)	*0.516*
Exercises			
6MWT, distance (m)	149.9 (112.4)	236.7 (112.8)	**0.049**
Muscle strength, time (s)	60 (48)	82 (47)	**0.094**

Bold values denote statistical significance

Data were collected in the afternoon. Data are mean (SD)

*m* male, *f* female, *BMI* body mass index, *pCO*_*2*_ capillary carbon dioxide pressure, *pO*_*2*_ capillary oxygen pressure, *6MWT* 6-min walking test, *VCmax* maximum vital capacity, *FVC* forced vital capacity, *FEV1* forced expiratory volume in 1 s, *TLC* total lung capacity, *RV* residual volume, *sReff* specific resistance, *Rtot* total resistance

The patients with deventilation syndrome reported more dyspnea at the BORG scale at baseline (mean: 3.9 ± 2.7) than the control group (2.1 ± 1.9; p = 0.042). There were no significant differences in the CAT questionnaire. In the SRI questionnaire, significant differences were reported in the summary score and the subcategories ‘respiratory complaints’ and ‘physical functioning’ (Table 2).

**Table 2:** Health related life quality

	Patients with deventilation syndrome (*n* = *11*)	Patient without deventilation syndrome (*n* = *20*)	*p*
Questionnaires			
Borg scale	3.9 (2.7)	2.1 (1.9)	**0.042**
CAT	31.7 (5.7)	28.1 (7.5)	0.144
SRI Summary Score	37.6 (10.1)	50.6 (16.7)	**0.027**
SRI Respiratory Complaints	38.4 (14.3)	56.5 (23.8)	**0.030**
SRI Physical Functioning	24.2 (17.5)	40.0 (18.5)	**0.029**
SRI Attendant Symptoms and Sleep	52.6 (19.6)	56.7 (22.6)	0.617
SRI Social Relationships	49.6 (17.8)	61.3 (18.6)	0.146
SRI Anxiety	37.3 (15.2)	50.8 (26.5)	0.133
SRI Psychological Well Being	35.9 (17.4)	48. 5 (21.1)	0.106
SRI Social Functioning	26.4 (19.0)	40.2 (20.6)	0.081

Bold values denote statistical significance

Data were collected in the afternoon. Data are mean (SD)

*SRI* Severe Respiratory Insufficiency Questionnaire, *CAT* COPD Assessment Test

### Changes during the deventilation syndrome

**Fig. 2:**
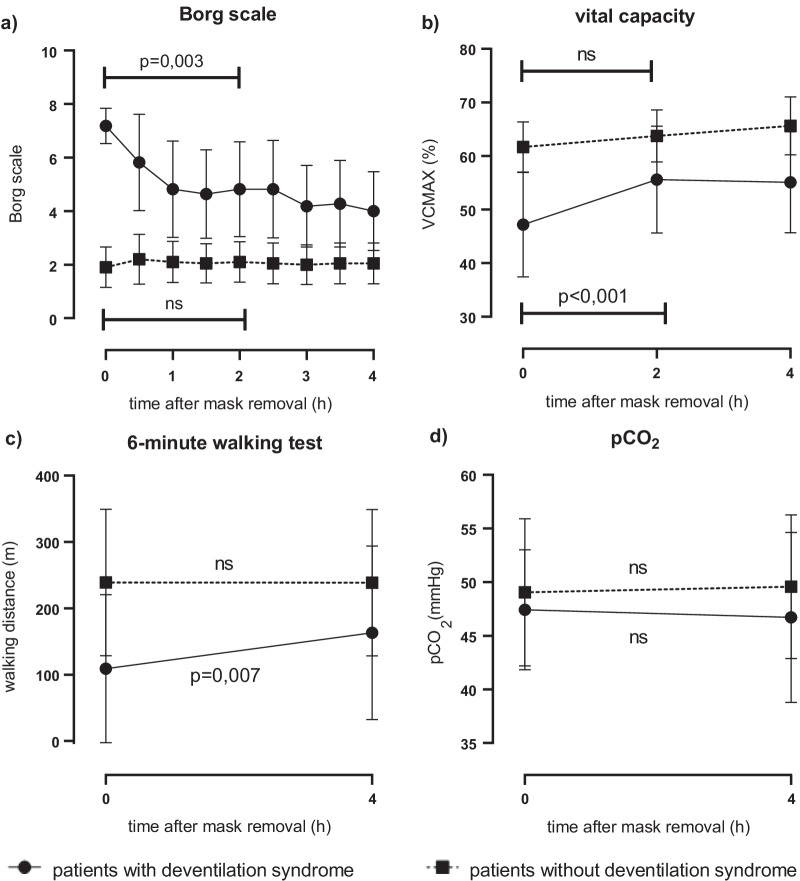
Measurements after ventilation in patients with deventilation syndrome compared to control group. Dots show mean values, bars show standard deviation. *VCmax*: maximum vital capacity, *pCO*_*2*_ capillary carbon dioxide partial pressure

Immediately after mask removal, the patients who reported a deventilation syndrome experienced dyspnea with a mean Borg scale of 7.2 ± 1.0, that decreased significantly to 4.0 ± 2.2 in the following hours (p < 0.001). The patients in the control group reported dyspnea of 1.9 ± 1.6 on the Borg scale after ventilation that remained stable in the next hours (2.1 ± 1.6 after 4 h; p = 0.5). The mean vital capacity of the patients with morning dyspnea was 46 ± 16% immediately after mask removal and 54 ± 15% after 2 h (p = 0.002). Between the second (2 h) and the third measurement (4 h), no changes were observed. In the control group, no significant changes were observed. The initial inspiratory flow did not change significantly. The deventilation syndrome patients achieved a mean walking distance of 109 ± 111 m in 6MWT immediately and 163 ± 131 m 4 h after mask removal (p = 0.007). In the control group, changes were non-significant. No significant changes in pCO_2_ were observed. There were also no changes and differences in oxygen saturation or heart rate in both groups immediately after ventilation. Detailed results are shown in Table 3 and Fig. 2.

**Table 3:** Deventilation syndrome

	Baseline (afternoon)	After mask removal	After 2 h	After 4 h	p*
Patients with deventilation syndrome (n = 11)					
Borg Scale	3.9 (2.7)	7.18 (0.98)	4.82 (2.64)	4.00 (2.19)	**0.003**
pCO_2_(mmHg)	47.0 (9.7)	47.42 (5.60)		46.71 (7.92)	0.492
6MWT walking distance (m)	149.9 (112.4)	109.00 (111.45)		163.09 (130.75)	**0.007**
VCmax (%)	51 (16.9)	46.00 (16.08)	54.86 (14.65)	54.55 (14.56)	** < 0.1**
Inspiratory flow (ml)	68.2 (16.6)	59.00 (19.12)	66.50 (13.55)	69.50 (12.13)	0.067
Patients without deventilation syndrome (n = 20)					
Borg Scale	2.1 (1.9)	1.9 (1.64)	2.10 (1.58)	2.05 (1.64)	0.330
pCO_2_(mmHg)	46.7 (7.0)	49.05 (6.85)		49.58 (6.70)	0.510
6MWT walking distance (m)	236.7 (112.8)	238.95 (110.33)		238.45 (110.36)	0.789
VCmax (%)	67 (12.2)	61.70 (10.04)	65.25 (12.98)	66.65 (11.17)	0.079
Inspiratory flow (ml)	72.0 (12.8)	68.25 (12.49)	69.75 (12.92)	71.00 (12.90)	0.316

Bold values denote statistical significance

Data are mean (SD)

*pCO*_*2*_ capillary carbon dioxide pressure, *6MWT* 6-min walking test, *VCmax* maximum vital capacity

*If available, values after mask removal were compared to measurement after 2 h otherwise to measurement after 4 h

The mean changes of both groups were compared. While the difference in change in Dyspnea (p ≤ 0.001) and 6-min walking test (p ≤ 0.001) were significant, the differences between patients with and without deventilation syndrome in vital capacity, PaCO_2_ and inspiratory flow were not. Details are shown in Table 4.

**Table 4:** Comparison of changes in both groups

	Patients with deventilation syndrome (n = 11)	Patients without deventilation syndrome (n = 20)	p
Δ After mask removal/2 h			
Δ Borg Scale	− 2.36 (2.063)	0.20 (0.894)	** < 0.001**
Δ VCmax (%)	7.76 (6.39)	3.55 (8.54)	0.144
Δ Inspiratory flow (ml)	7.50 (11.36)	1.58 (6.68)	0.087
Δ after mask removal/4 h			
Δ PaCO_2_ (mmHg)	− 0.71 (3.14)	0.53 (3.44)	0.340
Δ 6MWT walking distance (m)	54.09 (50.51)	− 0.5 (8.03)	** < 0.001**

Bold values denote statistical significance

Data are mean (SD)

*PaCO*_*2*_ capillary carbon dioxide pressure, *PaO*_*2*_ capillary oxygen pressure, *6MWT* 6-min walking test, *VCmax* maximum vital capacity

### Established NIV mode compared to PLBV mode

Compared to established treatment, the inspiratory pressure could be reduced from 17.5 ± 4.4 to 14.6 ± 3.0 mmHg in PLBV mode (p = 0.002) with no changes in blood gas analysis results. Reported dyspnea immediately after ventilation decreased significantly from 7.2 ± 1.0 to 5.6 ± 2.0 points on the Borg scale (p = 0.019). VCmax immediately after ventilation was higher on PLBV with 44.4 ± 16.0 versus 47.7 ± 16.2 (p = 0.040) with the established NIV. The walking distance in 6MWT after ventilation increased from 114.9 ± 115.7 m to 129.8 ± 132.2 m when treatment was switched to PLBV, but the difference was not significant (p = 0.087). Detailed results are shown in Table 5.

**Table 5:** Patients with deventilation syndrome under PLBV compared to established NIV

	Established NIV	PLBV mode	*p*
Ventilator settings			
Inspiratory pressure	17.5 (4.4)	14.6 (3.0)	**0.002**
Expiratory pressure	5.8 (1.1)	6.1 (1.3)	0.343
Borg scale	7.2 (1.0)	5.6 (2.0)	**0.019**
Blood gas analysis			
pH	7.419 (0.49)	7.418 (0.37)	0.849
pCO_2_(mmHg)	47.2 (5.9)	47.4 (6.3)	0.821
pO_2_ (mmHg)	64.6 (12.7)	67.9 (16.4)	0.326
BE (mmHg)	5.3 (5.5)	5.1 (4.8)	0.696
${\text{HCO}}_{3}^{ - }$ (mmHg)	30.5 (6.1)	30.4 (5.4)	0.873
6-min walking test			
Distance (m)	114.9 (115.7)	129.8 (132.2)	0.087
Pulmonary function			
VCmax (%)	44 (16.0)	48 (16.3)	**0.040**
FVC (%)	41 (19.0)	47 (18.4)	**0.032**
FEV1 (%)	20 (9.5)	21 (8.2)	0.415
FEV1/FVC (%)	37 (8.3)	34 (5.4)	0.051
Inspiratory flow (ml)	59.5 (19.2)	63.5 (15.6)	0.085

Bold values denote statistical significance. n = 10

Measurements immediately after mask removal after a single night in PLBV mode. Data are mean (SD)

*pCO*_*2*_ capillary carbon dioxide pressure, *pO*_*2*_ capillary oxygen pressure, *BE* base excess, ${\text{HCO}}_{3}^{ - }$ bicarbonate, *VCmax* maximum vital capacity, *FVC* forced vital capacity, *FEV1*: forced expiratory volume in 1 s

## Discussion

In this study, we describe parameters associated with early morning dyspnea after mask removal following nightly NIV. We focused on clinically relevant parameters used to describe pulmonary function and exercise tolerance as no concrete definition of the deventilation syndrome was available in the literature. Due to the large number of clinical parameters studied, we could gain an impression of changes during the deventilation syndrome. We observed that the patients who suffered from a deventilation syndrome in the morning had worse results in 6MWT, lung function and reported more severe dyspnea on the Borg scale at all times of the day. Together with the significantly higher airway resistance found in patients with a deventilation syndrome, our findings cannot prove but support the hypothesis that morning dyspnea after NIV-therapy is the result of dynamic hyperinflation during ventilation [7, 12]. COPD patients usually adapt a longer expiratory phase to achieve adequate expiration despite high airway resistance. During NIV therapy, they possibly inhale a volume that cannot be exhaled before the next inspiratory phase is triggered, especially in controlled modes with short expiratory time. This can lead to massive hyperinflation with an increase of intrinsic positive end expiratory pressure (PEEP). Especially a chronically weakened diaphragm can struggle in overcoming that, leading to dyspnea after NIV therapy. We could not find any differences in muscle strength that may suggest a generalized muscle weakness causing morning dyspnea. Furthermore, muscle weakness should have led to a decrease in inspiratory flow, which we did not find. The measurement of respiratory muscle strength may help to verify our measurements of peripheral muscle strength but is challenging to perform in this population.

The patients who suffered from a deventilation syndrome also reported poorer HRQoL in the SRI compared to the control group. In addition to the higher airway resistance, we also found an increased RV/TLC. This indicates that hyperinflation is not a problem confined to NIV periods but all day. This may explain the poorer exercise tolerance and dyspnea at any time during the day. It has been postulated, that NIV can reduce hyperinflation in COPD patients [13], but in a group of patients, who experience severe acute hyperinflation during and after NIV therapy, the therapy might also exacerbate persisting hyperinflation.

The gold standard to determine hyperinflation requires bodyplethysmography that is not available on the bedside. Thus, direct measurement of intrathoracic gas-volume (ITGV) was not possible in our patients who were investigated in their bed immediately after mask removal in the morning. Furthermore, the strongly impaired physical condition of most patients after mask removal only allowed bedside spirometry. Therefore, we can only hypothesize, that the decrease in vital capacity is caused by hyperinflation. Measurement of intrathoracic pressure to prove this hypothesis would again require invasive investigations. Interestingly, the patients of the control group also had an increase in vital capacity during the morning. The regain of vital capacity in this group was worse than in the patients with deventilation syndrome but not significant. Nevertheless, a hypothesis generated from this finding could be, that some patients perceive morning dyspnea because their drop in vital capacity is stronger in relation to their “daytime steady state vital capacity” that allows them to function on a low but stable level. If the morning drop is less, less dyspnea is perceived. However, the improvement in vital capacity did not differ statistically between both groups, and could be influenced by the use of inhaled medication in the morning after the first measurements and the early examination time. Further examinations with larger samples sizes are necessary to verify the differences between both groups.

Our observations in patients treated with PLBV also support the notion that hyperinflation is causing morning dyspnea in deventilation syndrome. After the patients were switched to PLBV, morning dyspnea decreased and this was associated with an increased inspiratory vital capacity. This confirms results of our previous retrospective study on PLBV [8]. It can be assumed that an increase in airway pressure during exhalation results in better airflow control allowing a harmonization of alveolar filling pressure and prevention of small airway collapse. In PLBV mode, no respiratory rate was adjusted and the inspiratory pressure was decreased in some patients who had very severe morning dyspnea. Capillary blood CO_2_ levels were unchanged compared to their established NIV. This suggests that PLBV may be a potential treatment to reduce morning dyspnea while maintaining stable CO_2_ levels. However, due to the small number of patients treated with PLBV, this study was not designed to evaluate therapeutic effects.

With a mean inspiratory pressure of 16.7 mmHg in our cohort, the mean pressure level was relatively low, probably due to a lack of tolerance of higher pressures by the patients. In our pathogenetic model, a higher inspiratory pressure would lead to an exacerbation of morning dyspnea, but we cannot prove this based on our data. In our study, dyspnea persisted for 1 h after removal of the ventilation mask. Accordingly, performance in the 6MWT was significantly worse immediately after ventilator mask removal compared to later exercise tests. Interestingly, no changes in blood gas analysis or pulse oximetry were observed, suggesting that the patients can cope with the resulting acute increase in respiratory workload and NIV therapy can effectively reduce CO_2_ despite the deventilation syndrome.

We are aware of the limitations of our study. The broad number of measurements and its small sample size only allow an exploration of the deventilation syndrome. The small sample size is due to the advanced morbidity of the patients and the decreasing inpatient numbers during the COVID-19 pandemic. A case number of 40 patients was initially planned but could not be achieved due to sustained service disruptions by the COVID-19 pandemic. Furthermore, the subjects were classified into “deventilation syndrome” and “no deventilation” syndrome according to their subjective assessments on the Borg scale. However, given the lack of a published definition of this disabling condition this approach appears to represent the real-life setting best. The number and type of examinations, especially lung function and 6MWT can be very exhausting for patients with ventilatory failure. As some patients were unable to complete the full set of diagnostic tests, smaller case numbers in some investigations had to be taken into account. The intervention (change of ventilation mode in the deventilation syndrome group) could not be blinded.

## Conclusions

The deventilation syndrome in COPD patients describes acute morning dyspnea after removal of the ventilator mask. In our cohort, a decrease in vital capacity and exercise tolerance after mask removal could be seen. Patients with high airway resistance are at increased risk of developing a deventilation syndrome. With a prevalence of 35% in our study, it is a common side effect in COPD patients undergoing NIV therapy. Adjusting high inspiratory pressures, as recommended in international guidelines [6], while maintaining patient comfort during ventilation remains a clinical challenge. Apart from optimizing ventilator settings [7], ventilation in PLBV mode may be a potential strategy to prevent morning dyspnea. Although a reduction of hypercapnia was achieved in our study despite the occurrence of the deventilation syndrome, the long-term effects of the deventilation syndrome remain unclear and should be further elucidated. Also, further studies with larger sample sizes are necessary to focus on individual parameters to validate our findings.

## Data Availability

The dataset used and analyzed during the current study are available from the corresponding author on reasonable request.

## Abbreviations

BE: Base excess
BMI: Body mass index
CAT: COPD assessment test
CO_2_: Carbon dioxide
COPD: Chronic obstructive pulmonary disease
DVS: Deventilation syndrome
HCO_3_-: Bicarbonate
FEV1: First second of forced expiration
FVC: Forced vital capacity
IGTV: Intrathoracic gas-volume
NIV: Non-invasive ventilation
O_2_: Oxygen
PEEP: Positive end-expiratory pressure
PLBD: Pursed lips breathing delay
PLBP: Pursed lips breathing pressure
PLBV: Pursed-lips-breathing-ventilation
Reff: Effective airway resistance
Rtot: Total airway resistance
SRI: Severe Respiratory Insufficiency Questionnaire
sReff: Specific effective airway resistance
VCmax: Maximum vital capacity
6MWT: 6-Minute walking test

## References

[1] World Health Organization. The top 10 causes of death. 2020. https://www.who.int/news-room/fact-sheets/detail/the-top-10-causes-of-death. Accessed 26 Mar 2021.

[2] Ergan B, Oczkowski S, Rochwerg B, Carlucci A, Chatwin M, Clini E, Elliott M, Gonzalez-Bermejo J, Hart N, Lujan M, Nasilowski J, Nava S, Pepin J, Pisani L, Storre JH, Wijkstra P, Tonia T, Boyd J, Scala R, Windisch W (2019). European Respiratory Society guidelines on long-term home non-invasive ventilation for management of COPD. Eur Respir J.

[3] Clini E, Sturani C, Rossi A, Viaggi S, Corrado A, Donner CF, Ambrosino N (2002). The Italian multicentre study on noninvasive ventilation in chronic obstructive pulmonary disease patients. Eur Respir J.

[4] Köhnlein T, Windisch W, Köhler D, Drabik A, Geiseler J, Hartl S, Karg O, Laier-Groeneveld G, Nava S, Schönhofer B, Schucher B, Wegschneider K, Criée C, Welte T (2014). Non-invasive positive pressure ventilation for the treatment of severe stable chronic obstructive pulmonary disease: a prospective, multicentre, randomised, controlled clinical trial. Lancet Respir Med.

[5] Duiverman ML, Wempe JB, Bladder G, Vonk JM, Zijlstra JG, Kerstjens HA, Wijkstra PJ (2011). Two-year home-based nocturnal noninvasive ventilation added to rehabilitation in chronic obstructive pulmonary disease patients: a randomized controlled trial. Respir Res.

[6] Dreher M, Storre JH, Schmoor C, Windisch W (2010). High-intensity versus low-intensity non-invasive ventilation in patients with stable hypercapnic COPD: a randomised crossover trial. Thorax.

[7] Adler D, Perrig S, Takahashi H, Espa F, Rodenstein D, Pépin JL, Janssens JP (2012). Polysomnography in stable COPD under non-invasive ventilation to reduce patient–ventilator asynchrony and morning breathlessness. Sleep Breath.

[8] Jünger C, Reimann M, Krabbe L, Gaede KI, Lange C, Herzmann C, Rüller S (2020). Non-invasive ventilation with pursed lips breathing mode for patients with COPD and hypercapnic respiratory failure: a retrospective analysis. PLoS ONE.

[9] Esquinas AM, Ucar ZZ, Kirakli C (2014). Deventilation syndrome in severe COPD patients during long-term noninvasive mechanical ventilation: poor sleep pattern, hyperinflation, or silent chronic muscular fatigue?. Sleep and Breathing.

[10] Windisch W, Freidel K, Schucher B, Baumann H, Wiebel M, Matthys H, Petermann F (2003). The Severe Respiratory Insufficiency (SRI) Questionnaire: a specific measure of health-related quality of life in patients receiving home mechanical ventilation. J Clin Epidemiol.

[11] Jones PW, Harding G, Berry P, Wiklund I, Chen W-H, Kline LN (2009). Development and first validation of the COPD Assessment Test. Eur Respir J.

[12] Lukácsovits J, Carlucci A, Hill N, Ceriana P, Pisani L, Schreiber A, Pierucci P, Losonczy G, Nava S (2012). Physiological changes during low- and high-intensity noninvasive ventilation. Eur Respir J.

[13] Budweiser S, Heinemann F, Fischer W, Dobroschke J, Pfeifer M (2005). Long-term reduction of hyperinflation in stable COPD by non-invasive nocturnal home ventilation. Respir Med.

